# Sub-Chronic Methomyl Exposure Induces Oxidative Stress and Inflammatory Responses in Zebrafish with Higher Female Susceptibility

**DOI:** 10.3390/antiox13070871

**Published:** 2024-07-20

**Authors:** Mingxiao Li, Xi Chen, Chao Song, Jing Xu, Limin Fan, Liping Qiu, Dandan Li, Huimin Xu, Shunlong Meng, Xiyan Mu, Bin Xia, Jun Ling

**Affiliations:** 1 Wuxi Fishery College, Nanjing Agricultural University, Wuxi 214081, China; 2023213004@stu.njau.edu.cn (M.L.); chenxi@ffrc.cn (X.C.); songchao@ffrc.cn (C.S.); fanlm@ffrc.cn (L.F.); 2 Freshwater Fisheries Research Center, Chinese Academy of Fishery Sciences, Scientific Observing and Experimental Station of Fishery Resources and Environment in the Lower Reaches of the Changjiang River, Wuxi 214081, China; qiuliping@ffrc.cn (L.Q.); lidandan@ffrc.cn (D.L.); xuhuimin@ffrc.cn (H.X.); 3 Environmental Testing Centre, Wuxi 214028, China; davida0006@sina.com; 4 Institute of Quality Standard and Testing Technology for Agro-Products, Chinese Academy of Agricultural Sciences, Beijing 100081, China; muxiyan@cafs.ac.cn; 5 Yellow Sea Fisheries Research Institute, Chinese Academy of Fishery Sciences, Qingdao 266071, China; xiabin@ysfri.ac.cn; 6 Fisheries Institute, Anhui Academy of Agriculture Sciences, Hefei 230031, China

**Keywords:** methomyl, oxidative stress, inflammation, sex differences, zebrafish

## Abstract

The widespread use of carbamate pesticides has raised significant environmental and health concerns, particularly regarding water contamination and the disruption of defense systems in organisms. Despite these concerns, research on the differential impacts of pesticides on male and female organisms remains limited. This study focused on methomyl, investigating sex-specific differences in liver antioxidant defenses and inflammatory response indices in male and female zebrafish after 56 days of exposure to environmentally relevant concentrations (0, 0.05, 0.10, and 0.20 mg/L). Our findings indicate that methomyl exposure significantly increased ROS content in zebrafish livers, inducing oxidative stress and activating enzymatic antioxidant defenses such as SOD, CAT, and GSH-Px activities. Sub-chronic exposure altered the expression of apoptosis-related genes (*Bax/Bcl2a* and *Caspases3a*), resulting in liver cell apoptosis in a concentration-dependent manner, with the 0.20 mg/L concentration causing the most severe damage. Additionally, methomyl exposure at environmentally relevant concentrations triggered persistent inflammatory responses in liver tissues, evidenced by increased transcription levels of inflammatory factor genes and the activation of toll-like receptors, heightening susceptibility to exogenous allergens. It is noteworthy that oxidative damage indicators (AST, ROS, MDA) and inflammatory gene expressions (*IL-1β*, *TNF-α*) were significantly higher in female livers compared to male livers at 0.10–0.20 mg/L methomyl exposure. Consequently, our study underscores the potential adverse effects of environmental methomyl exposure on aquatic organisms and highlights the need for heightened consideration of the risks posed by environmental endocrine disruptors to female health and safety.

## 1. Introduction

According to the Food and Agriculture Organization of the United Nations, global commodity production of primary crops is projected to reach 9.5 billion tons in 2021, representing an approximate 54% increase from the production levels in 2000 [1]. The use of pesticides, such as insecticides and herbicides, has played a significant role in achieving this outcome. However, it is estimated that only 10% of the pesticides applied actually affect the target crops, with the remaining 90% persisting in the environment and entering the ecological cycle, posing a potential threat to non-target ecosystems and human health [2].

Methomyl (*S*-methyl-N-[(methylcarbamoxyl) oxy] thioacetimidate, CAS 16752-77-5, C_5_H_10_N_2_O_2_S) is an oxime carbamate insecticide used to control a wide range of insect classes. It is highly hepatotoxic, cytotoxic, and neurotoxic, primarily by accumulating in organisms and inhibiting acetylcholinesterase (AChE) activity, leading to nerve and tissue failure. Despite regulations and restrictions in some countries, methomyl remains a problem due to its high water solubility (57.9 g/L at 25 °C), long half-life (surface water, 6 days; groundwater, 25 weeks), and weak to moderate soil adsorption [3,4]. Methomyl readily contaminates surface and groundwater, endangering ecological equilibrium and human health, particularly in areas of extensive use [5]. Of concern to the community is that exposure to pesticides like methomyl induces adverse outcomes such as endocrine disorders, cancer, diabetes, obesity, and cardiovascular diseases [6,7,8], for which the common pathogenic mechanisms include increased oxidative stress and persistent inflammation [9,10,11,12]. However, the influence of sex differences in organisms on the toxicological hazards of methomyl exposure remains largely unexplored.

Zebrafish (*Danio rerio*) is a recognized model organism [13] for toxicology studies due to its sensitivity to exogenous compounds, genetic similarity to humans, and disease characteristics, and zebrafish is widely used to explore the toxicity mechanisms of environmental pollutants [14,15]. The liver, a key organ for xenobiotic biotransformation, contains enzymes like alanine aminotransferase (ALT), aspartate aminotransferase (AST), and gamma-glutamyl transpeptidase (γ-GT), which are classical markers of liver damage from environmental toxicants [16,17,18]. Antioxidant enzymes such as superoxide dismutase (SOD), catalase (CAT), and glutathione peroxidase (GSH-Px) are the first line of defense against oxidative stress, and their significant increase in methomyl-exposed fish suggests a heightened primary defense [19,20]. In addition, inflammation is critical in defending and supporting the body following injury, trauma, and infection [21]. Recognition of various external stimuli by the innate immune system leads to the activation of multiple intracellular signaling pathways, including the NF-κB signaling pathway. NF-κB, a transcription factor involved in the sustained inflammatory response, regulates the expression of various pro-inflammatory genes and promotes the production of inflammatory factors (IL-1β, TNF-α, IL-6), serving as a key mediator of the inflammatory response [22]. Studies have documented that hepatic transcription factor expression and enzyme activities are regulated by sex [23,24], which may affect biological sensitivity to toxic contaminants, including methomyl. Recognizing these differences is, therefore, critical for accurately assessing toxicant risk and understanding the broader effects of toxicant exposure.

Therefore, we planned to expose zebrafish to environmental concentrations of methomyl over an extended period, analyzing the effects of exposure concentration and sex-specific differences on antioxidant enzyme activities, apoptosis, and the transcription levels of inflammation-related genes. The aim was to understand the sex-specific toxicological effects of methomyl exposure in zebrafish, provide critical insights for personalized medicine approaches, aid in the development of sex-specific guidelines for methomyl exposure limits, and establish a foundation for comprehensive and inclusive safety regulations.

## 2. Materials and Methods

### 2.1. Ethics Statement

The experimental procedures adhered to the guidelines set forth by the Ethics Committee at the Freshwater Fisheries Research Centre of the Chinese Academy of Fishery Sciences (FFRC, Wuxi, China) under approval number 2011AA1004020012.

### 2.2. Fish and Chemicals

Wild-type AB zebrafish lines were sourced from Shanghai Fauci Biotechnology Co. Ltd. (Shanghai, China) and acclimated to new environment in 100 × 50 × 60 cm glass tanks for one week. 

Methomyl (CAS 16752-77-5), with a purity of 97% (*w*/*w*), served as the chemical agent in our study. All reagents, which were sourced from Sigma-Aldrich (St. Louis, MO, USA) and Sangon Biotech (Shanghai, China), were of analytical reagent grade.

### 2.3. Experimental Design

A total of 800 juvenile zebrafish (body length 22.9 ± 1.66 mm, weight 0.20 ± 0.06 g) were randomly assigned to four concentration gradients of methomyl (0, 0.05, 0.10, and 0.20 mg/L), with four replicates for each treatment concentration (50 zebrafish/100 L methomyl/replicate). The selection of these concentration gradients was based on the residue level (0–0.097 mg/L) of methomyl in environmental water sources [25,26,27] and the safe concentration (0.212 mg/L) from the acute toxicity experiment of Meng [28]. 

To ensure the consistency of exposure concentrations, the methomyl solution was replaced with an equivalent volume of fresh solution every three days. Water samples were collected at 0 h (initial concentration) and 48 h post-exposure, then extracted and concentrated using solid-phase extraction (SPE). A Waters HLB extraction column was used for this purpose. The column was activated with 5 mL of methanol and 5 mL of pure water. The water samples were added to the column, which was subsequently eluted twice, first with 2 mL of methanol and then with 3 mL of methanol. The eluent was collected, shaken well, and diluted with methanol. The solution was then filtered using a 0.22 µm organic phase filter membrane and transferred into a 1.5 mL Waters sample vial. The actual concentration of methomyl was quantified using high-performance liquid chromatography–triple quadrupole mass spectrometry (HPLC-MS, Xevo TQ-S, Water, Germany), following the method described by Meng [19]. During the acclimatization period and sub-chronic exposure periods, zebrafish were fed twice daily (8:00 and 17:00) with *Artemia salina* eggs (crude protein ≥ 52%, crude fat ≥ 11%). The photoperiod was 14 h light/10 h dark, the water temperature was maintained at 26 ± 1 °C, the pH was maintained at 7.4 ± 0.2, and the dissolved oxygen levels were kept within the range of 6.5–7.0 mg/L.

### 2.4. Sample Collection

After 56 d of exposure, the fish were starved for 24 h and subsequently anesthetized using 100 mg/L MS-222 (Argent Chemical Laboratories, Redmond, WA, USA). From each replicate tank, six adult male and six adult female zebrafish were randomly selected and dissected to collect liver samples using a stereoscopic microscope (XTZ-AT, Shanghai, China). The liver tissues were rapidly frozen in liquid nitrogen and stored at −80 °C for subsequent enzyme activity measurements. Additionally, six adult male and six adult female zebrafish were randomly selected from each replicate tank for RNA extraction to assess gene expression changes, and the tissues were snap-frozen in liquid nitrogen and stored at −80 °C. Furthermore, liver tissues from six females and six males were collected from each concentration group and fixed in 4% paraformaldehyde for apoptosis staining to assess apoptotic activity.

### 2.5. Antioxidant Enzyme Activity Assay

The livers and pre-cooled phosphate-buffered saline (PBS) were mixed at a ratio of weight:volume = 1:19 before homogenization using a multi-sample tissue grinder (Tissuelyser-32L, Jingxin, Shanghai, China), and then centrifuged for 15 min at 3000× *g* and 4 °C (four liver tissues mixed into one sample, n = 6). The activities of ALT, AST, γ-GT, SOD, CAT, GSH-Px, and the contents of malondialdehyde (MDA) and reactive oxygen species (ROS) were examined in a multifunctional microplate reader (SpectraMax Mini, Molecular Devices, Silicon Valley, CA, USA) according to the instructions of Elisa kits. Additionally, the total protein (TP) content was determined using a biochemical kit according to the Bradford method. All kits were purchased from Shanghai Enzyme-linked Biotechnology Co., Ltd. (Shanghai, China).

### 2.6. Apoptosis Analysis

Fixed liver tissues were dehydrated, embedded, and sectioned into slices approximately 3 μm thick following the outlined protocol. Hepatic cell apoptosis was detected using a TUNEL (terminal dUTP nick-end labeling) kit (Yanjin Biotechnology Co., Ltd. Shanghai, China). The paraffin sections were immersed in xylene I (20 min), xylene II (20 min), and ethanol (100, 100, 85, 75%; 5 min at each concentration), and then washed with water for 2 min as described by Song et al. [29]. The tissues were covered with DNase-free proteinase K (20 μg/mL), incubated at 37 °C for 25 min, and washed with PBS three times for 5 min each time. Tissue samples were placed in a wet box, covered with TUNEL reaction solution, and incubated at 37 °C for 1 h. After incubating with 3,3-diaminobenzidine (DAB) for 5 min, sections were restained with hematoxylin for 12 s. The sections were dewaxed in xylene solution (Sinopharm, Shanghai, China) and then stained using TUNEL kits (Yanjin, Shanghai, China). The stained sections were observed and photographed under an ortho-fluorescent microscope (Eclipse C1, Nikon, Japan) equipped with an imaging system (DS-U3, Nikon, Japan). Images were analyzed using ImageJ software (v 1.54, National Institutes of Health, Silver Spring, Bethesda, MA, USA), with observations made in at least six different microscope fields per group.

### 2.7. Quantitative Real-Time PCR (qRT-PCR)

Gene expression analysis was conducted following the *Minimum Information Guidelines for Publication of Quantitative Real-Time PCR Experiments* (MIQE). Four liver samples were randomly selected as one sample, for a total of six samples for each concentration group. Total RNA was extracted using Trizol reagent (Invitrogen, Carlsbad, CA, USA), with the RNA concentration and quality assessed via a micro-spectrophotometer (Merinton SMA 4000, Ann Arbor, MI, USA) and 1% agarose gel, respectively. Approximately 500 ng of RNA (Abs 260/280 nm~2.0) was reverse-transcribed into cDNA using a HiScript III RT Super Mix kit (Vazyme, Nanjing, China). RT-qPCR analyses were performed on a CFX96™ Real-time PCR System (Bio-Rad, Hercules, CA, USA) using an AceQ^®^ qPCR SYBR^®^ Green Master Mix kit (Vazyme). Each reaction was performed in triplicate. *β-actin* mRNA served as a reference gene, with relative gene transcript levels calculated using the 2^−ΔΔCq^ method [29]. All primers were synthesized by Shanghai Sangon Biotechnology Co., Ltd. (Shanghai, China) and are detailed in Table 1.

### 2.8. Statistical Analysis

The number of apoptotic cells was analyzed using the Shapiro–Wilk test and Levene’s test, revealing that the data conformed to a normal distribution, but not to the principle of homogeneity of variance. Consequently, Welch’s test and Games–Howell multiple comparisons were used to determine the significance of these data. 

Other data from the exposed groups were presented as a ratio of the levels in the control group, referring to the study by Meng [30]. These data conformed to both normal distribution and homogeneity of variance, so we employed factorial (two-way) analysis of variance (ANOVA) with Bonferroni’s post hoc multiple comparisons correction to determine statistical significance. Significant interactions between the exposure concentration (C) and sex (S) warranted simple effect analysis to inspect treatment differences. A *p*-value < 0.05 was deemed statistically significant. Results are presented as mean ± standard deviation (SD) in bar charts.

Additionally, the significance of all raw data between the control and exposed groups was also analyzed using one-way analysis of variance (ANOVA) with Bonferroni’s post hoc multiple comparisons correction. These results are also expressed as mean ± standard deviation (SD) in a table, and statistical analyses were conducted using SPSS version 22.0 (SPSS Inc., Chicago, IL, USA).

## 3. Results

### 3.1. Actual Methomyl Concentrations

The initial methomyl concentrations in the groups designated as 0, 0.05, 0.10, and 0.20 mg/L were measured as 0, 0.057, 0.114, and 0.232 mg/L, respectively. After 48 h of exposure, these concentrations were recorded as 0, 0.049, 0.098, and 0.208 mg/L, respectively. This precise quantification validates the experimental conditions and ensures the scientific rigor and reliability of the subsequent findings, thus allowing the discussion and interpretation of the results to be based on the nominal concentrations with confidence.

### 3.2. Apoptosis of Zebrafish Livers

**Figure 1:**
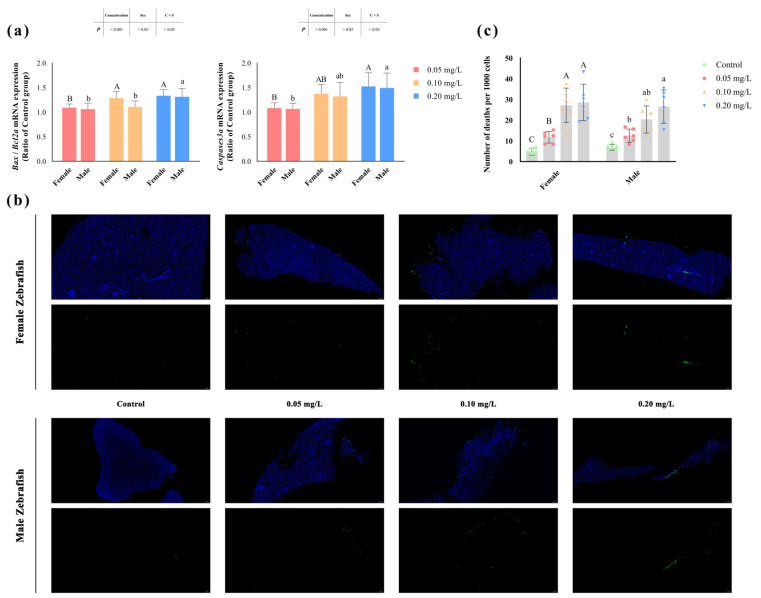
Apoptosis in the livers of male and female zebrafish after sub-chronic exposure to methomyl. (**a**) Changes in transcription levels of zebrafish hepatic *Bax/Bcl2a* and *Caspases3a* genes compared to the control group (expressed as ratios relative to the levels in the control group). (**b**) Representative images of TUNEL staining of liver sections from the control group and methomyl-exposed groups under a fluorescence microscope. The green dots represent dead cells, and the blue dots indicate live cells, scale bar: 50 μm. (**c**) Number of apoptotic cells observed per 1000 cells. Different letters indicate significant differences between concentration groups, *p* < 0.05.

The degree of apoptosis in zebrafish liver significantly increased (*p* < 0.05) after methomyl exposure (Figure 1b), and the number of apoptotic cells rose significantly as the concentration increased, reaching its highest value at 0.20 mg/L (Figure 1c). Similarly, the transcription levels of the *Bax/Bcl2a* and *Caspases3a* genes also showed a concentration-dependent increase (*p* < 0.05) (Table 2). However, no significant differences were observed between male and female zebrafish (*p* > 0.05) (Figure 1a).

### 3.3. Effects of Methomyl Exposure on Hepatic Antioxidant Defense System of Female and Male Zebrafish

**Figure 2:**
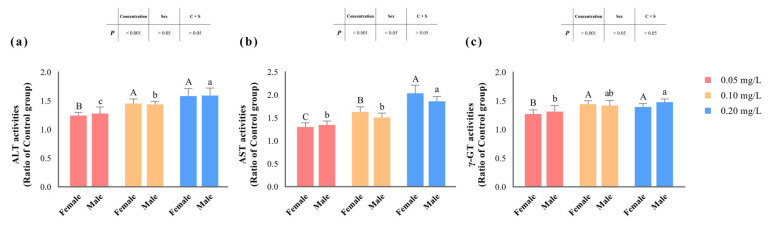
Changes in hepatic ALT (**a**), AST (**b**), and γ-GT (**c**) activities in zebrafish after sub-chronic exposure to methomyl compared to the control group (expressed as ratios relative to the levels in the control group). Different letters indicate significant differences between concentration groups, *p* < 0.05.

The activities of ALT, AST, and γ-GT significantly increased (*p* < 0.05) with rising concentrations of methomyl exposure compared to the control group, reaching their peak levels in the 0.20 mg/L-exposed group (Table 3, and Figure 2). However, there were no significant differences in the changes in ALT and γ-GT activities between male and female zebrafish (*p* > 0.05). Notably, only the changes in AST activity were significantly influenced (*p* < 0.05) by the sex of the zebrafish. Furthermore, none of the changes in enzyme activities exhibited an interaction effect between concentration and sex (*p* > 0.05).

**Figure 3:**
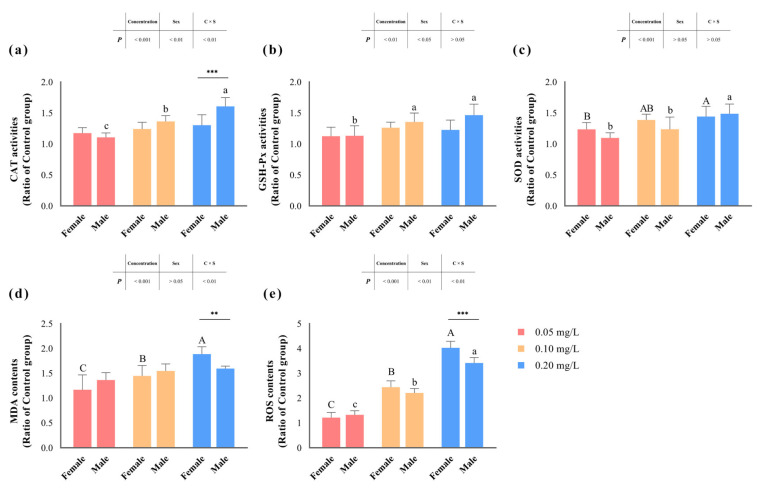
Changes in zebrafish hepatic CAT (**a**), GSH-Px (**b**), SOD (**c**), MDA (**d**), and ROS (**e**) levels after sub-chronic exposure to methomyl compared to the control group (expressed as ratios relative to the levels in the control group). Different letters indicate significant differences between concentration groups, *p* < 0.05. Asterisks (*) denote significant concentration–sex interaction effects for this concentration group. **: *p* < 0.01, ***: *p* < 0.001.

In our assessment of key enzyme activities within the antioxidant defense system, we observed significant effects (*p* < 0.05) of methomyl exposure concentration on the activities of CAT, SOD, and GSH-Px (Table 4 and Figure 3). Among them, the activities of CAT and GSH-Px displayed significant differentiation based on male/female sex (*p* < 0.05). Notably, CAT activity exhibited an interaction effect between concentration and sex (*p* < 0.05), with the maximum observed in the livers of male zebrafish exposed to 0.20 mg/L methomyl.

Similarly, hepatic MDA and ROS contents (expressed as a ratio of the control group) significantly increased (*p* < 0.05) with increasing methomyl exposure concentrations, showing a concentration–effect relationship. Interestingly, changes in MDA content did not show significant correlations with sex differences (*p* > 0.05), but were significantly influenced by the interaction effects of concentration and sex (*p* < 0.05). In addition, changes in ROS content were significantly impacted (*p* < 0.05), not only by exposure concentration and sex differences, but also by the interaction effect. Both MDA and ROS content peaked in the livers of female fish at the 0.20 mg/L exposure concentration.

### 3.4. Effects of Methomyl Exposure on Transcription Levels of Genes Associated with Hepatic Inflammation of Female and Male Zebrafish

**Figure 4:**
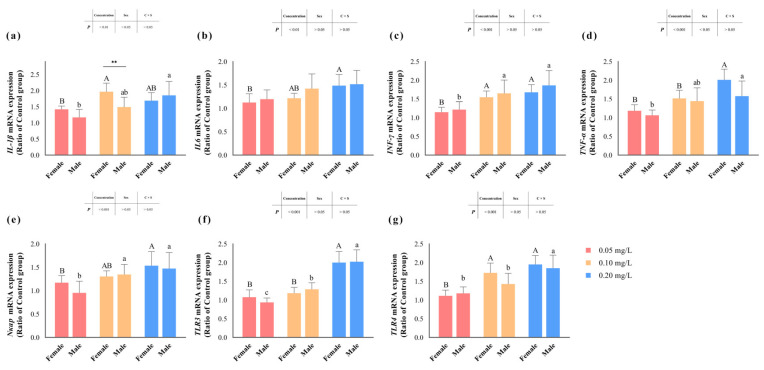
Changes in transcript levels of *IL-1β* (**a**), *IL6* (**b**), *INF-γ* (**c**), *TNF-α* (**d**), *Nκap* (**e**), *TLR3* (**f**), and *TLR4* (**g**) genes in zebrafish livers after sub-chronic exposure to methomyl compared to the control group (expressed as ratios relative to the levels in the control group). Different letters indicate significant differences between concentration groups, *p* < 0.05. Asterisks (*) denote significant concentration–sex interaction effects for this concentration group. **: *p* < 0.01.

Figure 4 illustrates the transcription levels of zebrafish liver inflammation-related genes after methomyl exposure. Genes encoding pro-inflammatory factors, such as *IL6*, *INF-γ* and *Nκap*, as well as toll-like receptors (TLRs) like *TLR3* and *TLR4*, displayed significant sensitivity to methomyl concentration (*p* < 0.05) (Table 2), showing an increasing trend with rising methomyl concentrations and reaching maximum levels in the group exposed to 0.20 mg/L methomyl. However, no discernible sex differences or interaction effects were observed in these genes (*p* > 0.05). The transcription levels of the *TNF-α* gene significantly increased (*p* < 0.05) compared to the control group, indicating a concentration–effect relationship. Interestingly, this alteration was significantly associated with sex, with females showing significantly higher levels compared to males (*p* < 0.05).

Additionally, the transcription levels of the *IL1β* gene demonstrated a distinctive pattern: while not significantly influenced by sex differences (*p* > 0.05), they were notably affected by methomyl concentration and the interaction effect of concentration and sex (*p* < 0.05). Specifically, females displayed significantly higher transcription levels compared to males at the exposure concentration of 0.10 mg/L. Overall, the transcription levels of the *IL1β* gene exhibited a significant and consistent increase in the livers of male zebrafish after methomyl exposure, contrasting with the increasing-then-decreasing trend observed in the livers of female zebrafish.

## 4. Discussion

Carbamate insecticides, such as methomyl, have been extensively utilized in agricultural pest control, resulting in their frequent detection in the environment. Previous studies have elucidated various detrimental effects of methomyl on organisms, including impacts on embryonic development [30], oxidative stress [31], apoptosis [32], energy metabolism [33], sex hormone secretion [34], immune response [35], and motor behavior [36]. However, little attention has been paid to whether there is a differential effect of methomyl on male and female fish. Our study seeks to address this knowledge gap by comparing the differences in hepatic antioxidant function, apoptosis, and inflammatory responses between male and female zebrafish after sub-chronic exposure to environmentally relevant concentrations of methomyl.

The liver serves as the primary organ for detoxification of xenobiotics and elimination of toxic substances in fish, but pollutants possess the potential to inflict structural damage upon it. SOD, CAT, and GSH-Px enzymes play crucial roles in the antioxidant defense system, and their activities are modulated by environmental pollution, thereby triggering the production of reactive oxygen species (ROS) and subsequent oxidative stress. In this experiment, we observed a substantial increase in ROS and MDA contents (utilized to evaluate cellular ROS oxidation) with escalating concentrations of methomyl exposure, peaking at the 0.20 mg/L exposure concentration. We also noted a pronounced concentration-by-sex interaction effect, with MDA and ROS alterations in the livers of females at the 0.20 mg/L concentration surpassing those in male fish. 

Furthermore, our study revealed an upward trend in CAT, SOD, and GSH-PX activities in the liver tissues of both female and male zebrafish after methomyl exposure, with significant alterations correlating with concentration. Simultaneously, CAT and GSH-Px activities were significantly influenced by the sex of zebrafish, with male zebrafish exhibiting higher activity levels than females at a concentration of 0.20 mg/L methomyl. Numerous studies on the toxic effects of pesticides such as diazinon [37], cypermethrin [38], atrazine [39,40], methomyl [41,42], quinalphos [43], lambda-cyhalothrin [44], carbosulfan [45], organochlorine pesticides (OCPs) [46], 2,4-dichlorophenoxyacetic acid, and azinphosmethyl [47] have documented similar results of induced oxidative stress in fish, including zebrafish, *Oreochromis Niloticus*, *Oncorhynchus mykiss,* and *Cyprinus Carpio*. Interestingly, differences in pesticide species, exposure concentrations, exposure durations, and exposure subjects may account for the varying outcomes in terms of changes in enzyme activities. For example, studies on diazinon [37] and cypermethrin [38] exposure have been shown to disrupt antioxidant defenses and reduce the activity of CAT, SOD, and GSH-Px enzymes in tissues, and exposure to methomyl, quinalphos [43], lambda-cyhalothrin [44], and atrazine [39] has been reported to induce the activation of antioxidant enzyme defenses, elevating their activities. These results ultimately suggest that exposure to pollutants elevates ROS levels, inducing oxidative stress in the organism and causing toxic damage. Therefore, based on this experiment, we concluded that chronic exposure to environmental concentrations of methomyl increased the ROS levels and MDA content in zebrafish livers, induced stress and oxidative damage, and activated the antioxidant defense system. Combined with the observation of lower ROS and MDA contents in the livers of male zebrafish compared to female zebrafish, we hypothesized that exposure to 0.20 mg/L methomyl posed a greater risk of oxidative toxicity injury to the livers of female zebrafish. 

ALT and AST are widely employed as conventional markers to evaluate liver function and injury, with their release indicating hepatocyte necrosis or membrane damage [48]. Additionally, γ-GT plays a pivotal role in maintaining glutathione homeostasis and oxidative stress balance by cleaving intact glutathione and transferring glutamyl to various molecular acceptors in ROS-exposed cells [49,50]. Our study revealed that hepatic ALT, AST, and γ-GT activities significantly increased after methomyl exposure, displaying a concentration–effect relationship. This finding is consistent with results from studies on the toxicity of pesticides such as methomyl [51], chlorpyrifos [52], glyphosate, and methidathion [53]. It is interesting to us that, when male and female zebrafish were separately exposed to methomyl, the increase in ALT activity was significantly higher in females than in males at a concentration of 0.20 mg/L methomyl. This suggests that methomyl exposure caused more pronounced liver damage in female fish. Moreover, methomyl induced apoptosis in zebrafish liver cells in a concentration-dependent manner, as evidenced by TUNEL staining and the transcriptional levels of *Bax/Bcl2a* and *Caspases3a*, which are important regulators in the apoptotic pathway [54].

ROS is widely acknowledged as a key mediator in necroptosis and can act in concert with the NF-κB pathway to regulate programmed cell death [55] and inflammatory responses [56] in animals. While the inflammatory response is vital for triggering the immune system’s defensive reaction against various danger signals [57], prolonged or chronic inflammation can lead to the release of proinflammatory cytokines (IL-1β, IL6, INF-γ, TNF-α), disrupting inflammatory processes’ delicate balance and resulting in severe symptoms and complications [58,59]. Additionally, TLRs play a crucial role in inflammatory and antiviral responses by sensing pathogen-associated molecular patterns (PAMPs) [60], with TLR3 and TLR4 being particularly sensitive to allergens and environmental pollutants [61,62], thereby activating the NF-κB pathway [63,64]. In our study, the transcription levels of the *IL-1β*, *IL6*, *INF-γ*, and *TNF-α* genes were significantly affected by methomyl concentration, as is consistent with exposure results for other pesticides such as Chlorpyrifos [10], Abamectin [12], oxyfluorfen [65], and organochlorine pesticides [66]. Similarly, the transcription levels of the *TLR3*, *TLR4*, and *Nkap* genes increased significantly after exposure, indicating that methomyl exposure induced an inflammatory response in zebrafish, likely related to increased susceptibility to allergens [67]. The data showed a clear concentration–effect relationship, with higher exposure concentrations correlating with greater toxicity hazards. Notably, *TNF-α* transcription levels were significantly correlated with sex differences, and *IL-1β* was significantly affected by the interaction effect of concentration and sex, especially in the 0.10 mg/L exposure group. The alterations in the transcription levels of these genes were significantly higher in females than in males, suggesting that female zebrafish exposed to environmental concentrations of methomyl may have a higher risk of chronic inflammation compared to male zebrafish.

## 5. Conclusions

**Figure 5:**
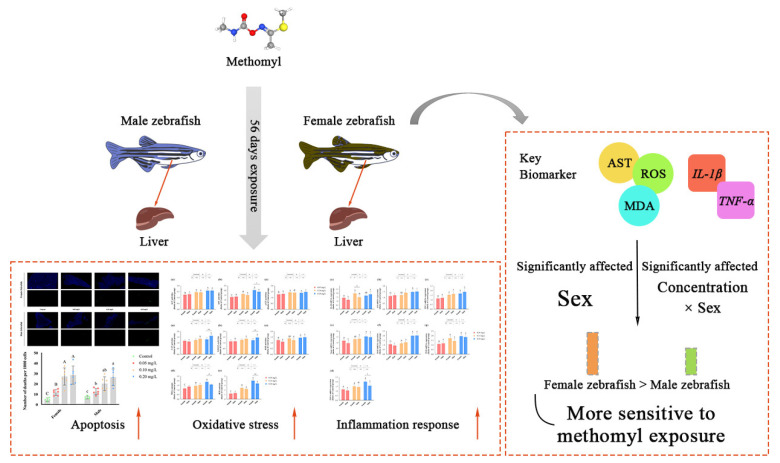
Summary of sub-chronic methomyl exposure in zebrafish livers: sex-susceptible biomarker responses. Key indicators such as AST, ROS, MDA, *IL-1β*, and *TNF-α* exhibited heightened sensitivity in females, suggesting that these markers can serve as effective biomarkers for assessing sex differences in response to methomyl exposure. Different letters indicate significant differences between concentration groups, *p* < 0.05, asterisks (*) denote significant concentration–sex interaction effects for this concentration group. **: *p* < 0.01, ***: *p* < 0.001.

The liver plays a pivotal role in the metabolism and detoxification of xenobiotics. Our study demonstrated that sub-chronic exposure to methomyl at concentrations of 0.10–0.20 mg/L for 56 days resulted in a significant increase in ROS levels in the zebrafish livers, accompanied by the activation of the antioxidant defense system. Methomyl exposure also upregulated the expression of apoptotic genes *Bax/Bcl2a* and *Caspases3a* and increased the number of apoptotic cells. This exposure-induced oxidative stress was accompanied by chronic low-grade inflammation, with genes encoding inflammatory factors being highly expressed at 0.10–0.20 mg/L exposure concentrations. This heightened inflammatory response may increase the susceptibility of zebrafish to pathogenic bacteria. In conclusion, the results demonstrate that methomyl exposure induced oxidative stress and inflammatory responses in zebrafish, and female zebrafish were more susceptible to the toxic effects of methomyl than male zebrafish (Figure 5). Furthermore, it is noteworthy that even concentrations of 0.10 to 0.20 mg/L, which are lower than the theoretical safe concentration derived from acute toxicity tests, were capable of inducing changes in liver transcription factors and enzyme activities after 56 days of sub-chronic exposure. Consequently, pesticide risk assessments should not solely rely on acute toxicity data, but must also consider the potential toxic effects arising from prolonged exposure.

The findings of our study underscore the imperative to develop sex-specific guidelines regarding exposure limits for pesticides such as methomyl and highlight the critical importance of integrating the toxic effects of long-term exposure into the pesticide risk assessment framework to ensure robust and protective measures for public health, addressing both acute and chronic toxicological impacts.

## Figures and Tables

**Table 1:** Sequences of primers used for RT-qPCR.

Gene Name	Abbreviation	mRNA Number	Sequence (5′–3′)
*Interleukin 1 beta*	*IL-1β*	NM_212844.2	F: GTGGACTTCGCAGCACAAAAR: CGTTCACTTCACGCTCTTGG
*Interleukin 6*	*IL6*	NM_001261449.1	F: GTGAAGACACTCAGAGACGAGCAGR: GGTTTGAGGAGAGGAGTGCTGATC
*Tumor necrosis factor alpha*	*TNF-α*	NM_212859.2	F: CGTCTGCTTCACGCTCCATAAGR: GTTAAATGGATGGCAGCCTTGG
*nuclear factor-kappaB activating protein*	*Nκap*	NM_001003414.1	F: TTTACTGCCAGGTGAAGGTGCR: TGACATAGCCAGACTTCTCAAACTC
*Interferon 1*	*IFN-γ*	NM_207640.1	F: GAATGGCTTGGCCGATACAGGATAR: TCCTCCACCTTTGACTTGTCCATC
*Toll-like receptor 3*	*TLR3*	NM_001013269.3	F: TGAGTTGGAGCATCACAGGGR: ACTTGTTGATGCCCATGCCT
*Toll-like receptor 4*	*TLR4*	XM_009307228.3	F: GAGAGCCATGCACTCGAATTAR: AACCGAGGAAGGGATACTGGA
*B-cell lymphoma2-associated X*	*Bax*	NM_131562.2	F: GTGTATGAGCGTGTTCGTCR: CGGCTGAAGATTAGAGTTGT
*B-cell lymphoma2 apoptosis regulator a*	*Bcl2a*	NM_001030253.2	F: TACTTTGCCTGTCGCCTTGTR: AGCGAGGAAAACTCCGACTG
*Caspase* *3, apoptosis-related cysteine peptidase a*	*Caspases3a*	NM_131877.3	F: AAAAGGGCTCGTTAAGCGGTR: GCCGATGTTGGGGTAGTTCA
*Beta-actin*	*β-actin*	NM_131031.2	F: GTACCCTGGCATTGCTGACR: CTGCTTGCTGATCCACATCTG

**Table 2:** Changes in hepatic gene transcription levels in male and female zebrafish after 56 days of methomyl exposure.

Methomyl Concentration		Control		0.05 mg/L		0.10 mg/L		0.20 mg/L	
		*Mean*	*SD*	*Mean*	*SD*	*Mean*	*SD*	*Mean*	*SD*
*Bax/Bcl2a* (mRNA)	*Female*	1.01 ^C^	0.12	1.10 ^BC^	0.08	1.29 ^AB^	0.14	1.34 ^A^	0.12
	*Male*	1.00 ^b^	0.09	1.07 ^b^	0.12	1.11 ^ab^	0.12	1.32 ^a^	0.17
*Caspases3a* (mRNA)	*Female*	1.00 ^C^	0.07	1.09 ^BC^	0.11	1.38 ^AB^	0.19	1.53 ^A^	0.28
	*Male*	1.01 ^b^	0.15	1.08 ^b^	0.12	1.34 ^ab^	0.29	1.51 ^a^	0.31
*IL-1β* (mRNA)	*Female*	1.01 ^C^	0.15	1.44 ^B^	0.10	1.99 ^A^	0.27	1.71 ^AB^	0.25
	*Male*	1.01 ^b^	0.19	1.19 ^b^	0.25	1.51 ^ab^	0.31	1.88 ^a^	0.44
*IL6* (mRNA)	*Female*	1.03 ^B^	0.25	1.15 ^B^	0.19	1.25 ^AB^	0.11	1.52 ^A^	0.25
	*Male*	1.01 ^b^	0.17	1.21 ^ab^	0.20	1.44 ^ab^	0.32	1.54 ^a^	0.30
*INF-γ* (mRNA)	*Female*	1.01 ^B^	0.13	1.16 ^B^	0.13	1.56 ^A^	0.17	1.69 ^A^	0.21
	*Male*	1.01 ^c^	0.17	1.23 ^bc^	0.21	1.68 ^ab^	0.36	1.89 ^a^	0.40
*TNF-α* (mRNA)	*Female*	1.01 ^C^	0.12	1.19 ^BC^	0.16	1.53 ^B^	0.22	2.02 ^A^	0.29
	*Male*	1.02 ^b^	0.22	1.09 ^b^	0.14	1.47 ^ab^	0.36	1.61 ^a^	0.42
*Nκap* (mRNA)	*Female*	1.00 ^B^	0.10	1.18 ^B^	0.15	1.31 ^AB^	0.12	1.54 ^A^	0.30
	*Male*	1.01 ^b^	0.13	0.96 ^b^	0.25	1.36 ^ab^	0.22	1.48 ^a^	0.34
*TLR3* (mRNA)	*Female*	1.01 ^B^	0.11	1.08 ^B^	0.19	1.19 ^B^	0.16	2.01 ^A^	0.30
	*Male*	1.02 ^b^	0.20	0.95 ^b^	0.12	1.31 ^b^	0.18	2.06 ^a^	0.32
*TLR4* (mRNA)	*Female*	1.01 ^B^	0.15	1.12 ^B^	0.16	1.75 ^A^	0.26	1.97 ^A^	0.24
	*Male*	1.01 ^c^	0.16	1.19 ^bc^	0.17	1.45 ^ab^	0.29	1.87 ^a^	0.35

Note. Different letters indicate significant differences between concentration groups (*p* < 0.05).

**Table 3:** Changes in liver function indices in male and female zebrafish after 56 days of methomyl exposure.

Methomyl Concentration		Control		0.05 mg/L		0.10 mg/L		0.20 mg/L	
		*Mean*	*SD*	*Mean*	*SD*	*Mean*	*SD*	*Mean*	*SD*
ALT (U/g prot)	*Female*	11.99 ^C^	1.36	14.92 ^B^	0.69	17.43 ^A^	0.97	18.99 ^A^	1.57
	*Male*	20.64 ^c^	0.77	26.39 ^b^	2.40	29.72 ^a^	1.02	32.89 ^a^	2.69
AST (U/g prot)	*Female*	11.01 ^D^	0.68	14.33 ^C^	1.01	17.96 ^B^	1.21	22.43 ^A^	1.94
	*Male*	17.99 ^c^	1.90	24.24 ^b^	1.50	27.15 ^b^	1.72	33.47 ^a^	1.88
γ-GT (IU/g prot)	*Female*	14.41 ^C^	1.50	18.33 ^B^	1.05	20.82 ^A^	0.83	20.11 ^AB^	0.84
	*Male*	21.99 ^c^	2.25	28.91 ^b^	2.31	31.24 ^ab^	1.94	32.53 ^a^	1.21

Note. Different letters indicate significant differences between concentration groups (*p* < 0.05).

**Table 4:** Changes in hepatic oxidative stress levels in male and female zebrafish after 56 days of methomyl exposure.

Methomyl Concentration		Control		0.05 mg/L		0.10 mg/L		0.20 mg/L	
		*Mean*	*SD*	*Mean*	*SD*	*Mean*	*SD*	*Mean*	*SD*
CAT (U/mg prot)	*Female*	15.14 ^C^	1.74	17.81 ^ABC^	1.33	18.81 ^AB^	1.64	19.74 ^A^	2.54
	*Male*	17.89 ^c^	2.78	19.82 ^c^	1.28	24.41 ^b^	1.69	28.78 ^a^	2.54
SOD (U/mg prot)	*Female*	34.81 ^C^	3.02	43.03 ^B^	3.81	48.29 ^AB^	3.29	50.21 ^A^	5.75
	*Male*	50.42 ^b^	9.57	55.33 ^b^	4.17	62.39 ^ab^	9.91	75.02 ^a^	7.91
GSH-Px (U/mg prot)	*Female*	86.65 ^C^	6.30	97.51 ^ABC^	12.62	109.39 ^A^	7.86	106.43 ^AB^	13.71
	*Male*	112.13 ^c^	16.19	126.99 ^bc^	18.13	152.02 ^ab^	16.16	164.45 ^a^	19.65
ROS (ng/mg tissue)	*Female*	9.50 ^C^	1.09	11.49 ^C^	1.97	23.18 ^B^	2.39	38.26 ^A^	2.50
	*Male*	18.62 ^d^	2.02	24.63 ^c^	3.09	41.06 ^b^	3.34	63.58 ^a^	4.07
MDA (nmol/mg tissue)	*Female*	0.35 ^C^	0.05	0.41 ^BC^	0.11	0.51 ^B^	0.07	0.67 ^A^	0.05
	*Male*	0.66 ^c^	0.10	0.90 ^b^	0.10	1.02 ^ab^	0.09	1.05 ^a^	0.03

Note. Different letters indicate significant differences between concentration groups (*p* < 0.05).

## Data Availability

Data is contained within the article.

## Abbreviations

Acetylcholinesterase, AChE; Alanine aminotransferase, ALT; Aspartate aminotransferase, AST; Gamma-glutamyl transpeptidase, γ-GT; Superoxide dismutase, SOD; Catalase, CAT; Glutathione peroxidase, GSH-Px; Malondialdehyde, MDA; Reactive oxygen species, ROS; Total protein, TP; Interleukin 1 beta, IL-1β; Interleukin 6, IL6; Tumor necrosis factor alpha, TNF-α; Nuclear factor-kappaB activating protein, Nκap; Interferon 1, IFN-γ; Toll-like receptor 3, TLR3; Toll-like receptor 4, TLR4; B-cell lymphoma2 associated X, Bax; B-cell lymphoma2 apoptosis regulator a, Bcl2a; Caspase3, apoptosis-related cysteine peptidase a, Caspases3a; Beta-actin, β-actin.
